# EXAMINING CHANGES IN STUDENTS' ATTITUDES TOWARD WORKING WITH OLDER ADULTS

**DOI:** 10.1093/geroni/igac059.2439

**Published:** 2022-12-20

**Authors:** Christopher O'Brien

**Affiliations:** Chatham University, Pittsburgh, Pennsylvania, United States

## Abstract

Curricular intervention studies have examined if instruction in aging and gerontology affects undergraduates’ attitudes, knowledge, and perceptions towards older adults. However, less is known about curricular impact on undergraduates’ intentions to work with older adults. By identifying factors that increase undergraduates’ intentions to work with older adults we may elucidate meaningful points of intervention to enhance pursuit of careers in the geriatric workforce. The current study examined baseline data from a longitudinal study examining the impact of an upper-level adult development psychology course on student attitudes towards working with older adults. It was hypothesized that there would be positive associations between attitudes towards working with older adults, knowledge about aging, and positive attitudes towards older adults. Participants were 19 undergraduate students enrolled in upper-level undergraduate psychology courses. Participants completed validated, self-report questionnaires related to their attitudes towards working with older adults, ageism attitudes, and attitudes and knowledge about aging. Bivariate correlation analyses were used to examine cross-sectional associations among main outcome variables. More positive explicit attitudes towards older adults were significantly associated with more willingness to work with older adults (r= .49 , p=.04). Additionally, knowledge of aging was positively correlated with perceived social norms around working with older adults (r= .49, p=.04). These initial findings suggest that knowledge and positive attitudes about aging may positively impact attitudes towards working with older adults. Future work will assess curricular impact on undergraduates’ intentions to work with older adults, as well as evaluate predictors of change in intentions.

